# Management of anesthesia and complications in children with Tracheobronchial Foreign Body Aspiration

**DOI:** 10.12669/pjms.35.6.1225

**Published:** 2019

**Authors:** Erol Karaaslan, Turan Yildiz

**Affiliations:** 1 Dr. Erol Karaaslan, Assistant Professor, Dept. of Anesthesiology and Reanimation, Inonu University, Medical Faculty, Malatya, Turkey; 2 Dr. Turan Yildiz, Associate Professor, Dept. of Pediatric Surgery, Inonu University, Medical Faculty, Malatya, Turkey

**Keywords:** Anesthetic management, Bronchoscopy, Complications, Intensive care, Tracheobronchial foreign body

## Abstract

**Objectives:**

Delayed diagnosis and treatment of tracheobronchial foreign body aspiration (FBA) in children may lead to morbidity and mortality. Our objective was to evaluate the anesthetic management, peri- and post-operative complications, and predisposing factors for postoperative intensive care unit (ICU) admission in children undergoing rigid bronchoscopy due to tracheobronchial FBA.

**Methods:**

This retrospective study included 81 children who underwent rigid bronchoscopy between January 2010 to July 2018 at Inonu University, Department of Pediatric Surgery, Turkey. Data regarding demographic characteristics, anesthetic management, length of ICU and hospital stays, and peri- and post-operative complications were retrieved from the hospital database.

**Results:**

The patients included 54 (66.7%) boys and 27 (33.3%) girls with a mean age of 29.6 ± 31.2 months. The most common presenting symptom was the suspicion of FBA, followed by acute-onset cough, cyanosis, wheezing and respiratory distress. Mean duration of anesthesia was 44.40 ± 14.72 min. Of the 81 patients, 18 (22.2%) were transferred to ICU and 63 (77.8%) patients were transferred to the ward postoperatively. Of the patients transferred to the ICU, 5 of them required mechanical ventilation. Some peri and postoperative complications, desaturation (n=16; 19.7%; p=0.001), mucosal bleeding (n=6; 7.4%; p=0.02), laryngeal edema (n=11; 13.6%; p<0.001), laryngospasm (n=13; 16.3%; p<0.001), were affected the frequency of intensive care transfer.

**Conclusion:**

Bronchoscopy with general anesthesia remains the golden standard for the management of tracheobronchial FBA. In such patients, a combination consisting of a detailed preoperative clinical evaluation of the patient, selection of short-acting anesthetic agents with minimal side effects for the induction and maintenance of anesthesia, and the administration of controlled ventilation can be recommended. Additionally, early diagnosis of peri- and post-operative complications, prediction of postoperative ICU requirement, and a close cooperation of anesthesiologists and surgeons are highly important.

## INTRODUCTION

Tracheobronchial foreign body aspiration (FBA) is the aspiration of a foreign body (FB) into the tracheobronchial tree through the mouth or nose during inhalation and is the most common cause of acute-onset upper respiratory airway obstruction. Approximately 80% of tracheobronchial FBA cases are seen in children aged 0-3 years and tracheobronchial FBA is responsible for 0-1.8% of deaths in this age group. Moreover, FBA is the most common cause of accidental deaths in children less than one-year-old.^1^,^2^

Bronchoscopy is the standard treatment modality for confirming the presence of a tracheobronchial FB in children and removing it. Rigid bronchoscope is mostly used for the removal of solid FBs. Undiagnosed and retained FBs may lead to serious complications such as pneumonia, lung abscess, and bronchiectasis.^2^,^3^ Rigid bronchoscopies is safely performed under general anesthesia in an operating room environment. Given the shared use of the airway by the surgeon and the anesthesiologist, bronchoscopy requires experienced teams for securing sufficient ventilation and oxygenation in the patient.^4^ Anesthetic managements during bronchoscopic procedures, particularly the anesthetic technique to be used, is of prime importance due to the risk of peri- and post-operative complications. Successful anesthetic management and removal of an aspirated FB has a contributory effect on the reduction of morbidity and mortality.^5^,^6^

The aim of this study was to evaluate the demographic characteristics, anesthetic management, peri and post-operative complications, and predisposing factors for postoperative intensive care unit (ICU) admission in children undergoing rigid bronchoscopy due to tracheobronchial FBA.

## METHODS

This retrospective study included 95 children who were diagnosed with tracheobronchial FBA by a combination of patient history, physical examination, and radiological findings and underwent emergency or elective rigid bronchoscopy in Inonu University, Department of Pediatric Surgery between January 2010 to July 2018. This trial was approved by the Local Ethic Committee of Inonu University (Protocol no: 2019/3-15). Of these, 14 patients were excluded from the study and thus a total of 81 patients were included in the study. The study was conducted according to CONSORT guidance.^7^

The study included pediatric patients aged below 18 years with an American Society of Anesthesiologists (ASA) score of 1-4 who underwent bronchoscopy due to tracheobronchial FBA. Patients that did not undergo preoperative anesthetic evaluation, and had incomplete medical records.

### Preoperative Procedures

Premedication was performed with midazolam in all the patients except for the patients with respiratory distress. Following the transfer of the patients to the operating room, standard routine monitoring including noninvasive blood pressure (NIBP), heart rate (HR), peripheral oxygen saturation (SpO_2_), end-tidal carbon dioxide (EtCO_2_), and electrocardiogram was performed.

Following preoxygenation with four L/minutes 100% O_2_ for at least three minutes, general anesthesia was induced with appropriate combinations of propofol (1-2 mg.kg-1) or thiopental sodium (5 mg.kg-1), fentanyl (0.5-1 mcg.kg-1) or remifentanil (0.1-0.25 mcg/kg/min), succinylcholine (1 mg.kg-1) or rocuronium (0.5 mg/kg) at appropriate clinical doses of intravenous (i.v.) anesthetic agents. Anesthetic maintenance was achieved with sevoflurane MAC one in a 50:50 oxygen-air mixture. Reversal of neuromuscular blockade was achieved with neostigmine (0.05), atropine (0.02), or sugammadex (2 mg.kg-1). Intermittent positive-pressure ventilation was delivered via a side port of a rigid bronchoscope.

Following the bronchoscopy procedure, the patients were transferred to the post-anesthesia care unit (PACU) with ventilation provided either via bag-valve-mask or insertion of an orotracheal tube, as needed. The patients ventilated via bag-valve-mask were transferred to the ward when they achieved a Modified Aldrete’s score of 9 or greater (a score of nine on a 0-12 scale indicates recovery sufficient for the patient to be transferred from PACU to the ward)^8^. The patients with orotracheal intubation were transferred to the ICU. Postoperative analgesia was achieved with paracetamol (20 mg/kg, i.v) in all the patients.

Demographic characteristics, anesthetic management, presenting symptoms (suspicion of FBA, acute-onset persistent cough, cyanosis, wheezing, respiratory distress, nausea, vomiting, pneumonia), durations of anesthesia and surgery, peri- and post-operative complications (laryngeal edema, laryngospasm, bronchospasm, bradycardia, desaturation, mucosal bleeding, cough, nausea, vomiting, aspiration, cardiac arrest, and death), type and localization of aspirated FBs, length of ICU and hospital stays, and mortality were recorded for each patient.

Duration of anesthesia was defined as the time period from the transfer of the patient to the operating room to the transfer of the patient to PACU. Duration of surgery was defined as the time period from the initial introduction of the rigid bronchoscope tip in the patient’s mouth to the removal of the bronchoscope from the vocal cords. Laryngospasm was defined as glottal closure caused by the reactions obstructing ventilation of the lungs. Bronchospasm was defined as a prolonged expiratory phase accompanied by wheezing and desaturation. Bradycardia was defined as a resting heart rate of below 60 beats per minute. Desaturation was defined as a SpO2 level of 94% or below for more than 15 sec. Mucosal bleeding was defined as the presence of bleeding associated with the bronchoscopy procedure. Length of ICU stay was defined as the time period from the transfer of the patient to the ICU to the transfer of the patient to the ward postoperatively. Length of hospital stay was defined as the time period from hospital admission due to the suspicion of FBA to hospital discharge. Mortality was defined as death occurring in association with anesthesia or surgery during the hospital stay.

Quantitative variables were expressed as mean ± standard deviation (SD) and qualitative variables were expressed as frequencies and percentages. Qualitative data were analyzed with Yates corrected chi-square or Fisher’s exact test as appropriate. P<0.05 values were considered as significant. IBM SPSS Statistics version 25.0 for Windows was used for statistical analysis.

## RESULTS

The 81 patients included 54 (66.7%) boys and 27 (33.3%) girls with a mean age of 29.6 ± 31.2 months. The most common presenting symptom was the suspicion of FBA, followed by acute-onset cough, cyanosis, wheezing, respiratory distress, nausea-vomiting, and pneumonia. The ASA score was I in 18 (22.2%), II in 62 (76.5%), and III in 1 (1.3%) patient. Mallampati score was I in 71 (87.7% and II in 10 (12.3%) patients (Table-I).

**Table I:** Demographics and procedure data

	n (%) or Mean±std
Age, month	29.06 ± 31.20
***Gender, n***	
Male	54 (66.7)
Female	27 (33.3)
Weight, kg	25.59 ± 12.97
***ASA, n (%)***	
I	18 (22.2)
II	62 (76.5)
III	1 (1.2)
IV	0 (0)
***Mallampati***	
I	71 (88.8)
II	10 (12.3)
III	0 (%)
Duration of Anesthesia, min	44.30 ± 14.72
Duration of Surgery, min	27,35 ± 14.20
Length of Hospital Stay (LOS), day	2.25 ± 1.69
Length of ICU, day	0.65 ± 1.29
***Localization of Foreign Body (FB)***	
FB in right bronchial tree, n (%)	35 (43.3)
FB in left bronchial tree, n (%)	16 (19.7)
FB in larynx and trachea, n (%)	20 (24.6)
No FB	10 (12.3)

The data is presented as percentage or number of cases n: number of cases.

General anesthesia was induced with sodium thiopental in 5 (6.1%), propofol in 76 (93.8%), remifentanil in 64 (76%), and fentanyl in 17 (20.7%) patients. Neuromuscular blockade was achieved with rocuronium in 71 (87.7%) and succinylcholine in 10 (12.3%) patients. Sevoflurane was used for anesthetic maintenance in all the patients (100%). Reversal of neuromuscular blockade was achieved with sugammadex in 43 (60.5%) and with neostigmine-atropine in 28 (39.5%) patients. Prednol one mg/kg was administered in all the patients intraoperatively. Mean duration of anesthesia was 44.40 ± 14.72 min and mean duration of surgery was 27.35 ± 14.20 min. Perioperative complications included desaturation (n=16; 19.7%), bradycardia (n=8; 9.8%), mucosal bleeding (n=6; 7.4%), laryngeal edema (n=11; 13.6%), laryngospasm (n=13; 16.3%), and bronchospasm (n=4; 4.6%). The incidence of laryngeal edema, laryngospasm, desaturation, and mucosal bleeding was significantly higher in the patients transferred to the ICU postoperatively (*p*<0.05) (Table-II).

**Table II:** Peri- and postoperative complications.

Complications		Postoperative service of patients				P

		Ward		ICU		

		n	%	n	%	
Laryngeal edema	Absent	60	85.7	10	14.3	<0.001*
	Present	3	27.3	8	72.7	
Laryngospasm	Absent	58	86.6	9	13.4	<0.001*
	Present	4	30.8	9	69.2	
Bronchospasm	Absent	61	79.2	16	20.8	0.212*
	Present	2	50.0	2	50.0	
Desaturation	Absent	56	86.2	9	13.8	0.001*
	Present	7	43.8	9	56.3	
Mucosal Bleeding	Absent	61	81.3	14	18.7	0.02*
	Present	2	33.3	4	66.7	
Coughing	Absent	45	83.3	9	16.7	0.156^#^
	Present	18	66.7	9	33.3	
Nausea	Absent	55	77.5	16	22.5	0.61*
	Present	8	80.0	2	20.0	
Vomiting	Absent	60	80.0	15	20.0	0.120*
	Present	3	50.0	3	50.0	
Bradycardia	Absent	59	80.8	14	19.2	0.068*
	Present	4	50.0	4	50.0	
Death	Absent	63	100	18	100	
	Present	0	0	0	0	

* Fisher’s exact test; #: Yates corrected chi-square test.

Foreign bodies (FBs) were successfully extracted in 71 (87.7%) patients and no FBs were detected in the remaining 10 (12.3%) patients. The extracted FBs were mostly localized in the right main bronchus (n=35; 43.3%), followed by the larynx/trachea (n=20; 24.6%) and the left main bronchus (n=16; 19.7%). No patient had a FB lodged in both bronchi.

Of the 81 patients, 18 (22.2%) were transferred to the ICU postoperatively, five of whom required mechanical ventilation. Twenty-five (30.8%) patients with no acute respiratory distress were pre-medicated with midazolam and eight of them required postoperative ICU admission. Of the 18 patients transferred to the ICU postoperatively, 12 (66.7%) patients had organic and 4 (22.2%) patients had inorganic FBs, whereas 2 (11.1%) patients were detected with no FBs. Of the 16 FBs extracted in the patients transferred to the ICU, 8 (44.4%) of them were localized in the right bronchus, 6 (33.3%) in the left bronchus, and 2 (11.1%) in the trachea-larynx. Mean length of ICU and hospital stays were 0.65 ± 1.29 and 2.25 ± 1.29 days for 81 patients, respectively.

Of the 81 patients, 63 (77.8%) of them were transferred to the ward postoperatively, among whom supplemental oxygen was administered via nasal cannula in 50 (79.4%), nausea occurred in 10 (12.3%), and vomiting occurred in 6 (7.4%) patients. No signs of aspiration were observed in any patient peri- or post-operatively.

## DISCUSSION

Most of tracheobronchial FBA cases are seen in children aged 1-3 years, predominantly in boys. Children in this age group are particularly susceptible to FBA due to the propensity to insert objects in their mouth, lack of molars leading to ineffective chewing, and their tendency to play, laugh, and move around while eating.^5^,^9^,^10^ As consistent with the literature, most of the patients in our study were aged below three years and the incidence of FBA was higher in boys compared to girls.

Literature indicates that there is no consensus on the anesthetic technique to be used in the management of tracheobronchial FBA in children. In such patients, selection of the anesthetic technique and agents is based on the ventilation technique planned for each patient. These ventilation techniques include spontaneous and controlled ventilation, both of which have been shown to have both advantages and disadvantages. Liu et al. compared spontaneous and controlled ventilation and reported that there was no difference between the two techniques with regard to desaturation while the incidence of laryngospasm was higher in the patients that underwent spontaneous ventilation.^11^ However, in a previous retrospective study, Chen et al. reported that spontaneous ventilation led to a greater incidence of intraoperative body movement, straining, laryngospasm, and hypoxia and significantly longer duration of emergence from anesthesia compared to other ventilation techniques.^12^

A controlled ventilation technique, consisting of administration of neuromuscular blockade, has been shown to have several advantages such as prevention of body movement and straining, increased anesthetic depth, easy passage of the bronchoscope through the vocal cords, and reduction of the incidence of airway obstruction and atelectasis.^6^,^13^ However, controlled ventilation can be disadvantageous as well since it may require additional positive-pressure breaths, thereby leading to unintentional movement of the FB further distally.^13^,^14^ In our study, we also performed controlled positive-pressure ventilation in our patients.

Selection of anesthetic agents for a surgical procedure also depends on the personal knowledge and experience of anesthesiologists, with propofol and sodium thiopental reported to be the most common agents for anesthetic induction.^15^ In our study, propofol was administered in all the patients.

There are numerous studies in the literature reporting on the use and advantages of sevoflurane in the induction and maintenance of anesthesia in children undergoing bronchoscopy due to tracheobronchial FBA. Accordingly, sevoflurane has been shown to be advantageous over other agents since it is relatively less irritant, facilitates the removal of the FB by reducing the airway resistance, and has fewer side effects.^16^ Nevertheless, sevoflurane has been reported to have several disadvantages as well, such as requirement of higher sevoflurane concentrations for the achievement of a deep level of anesthesia during bronchoscopy due to the air leakage around the rigid bronchoscope which leads to pollution of ambient air.^17^ As consistent with the literature, we also used sevoflurane as the inhalation agent in all the patients.

Remifentanil, which is known to have a short half-life, has been shown to be effective in the prevention of hemodynamic response to laryngoscopy.^18^ Additionally, besides its analgesic effects, remifentanil has also been reported to have antitussive effects and favorable effects on cough.^19^ Previous studies reported that patients anesthetized with remifentanil without the administration of neuromuscular blockade had a lower incidence of cough while they had a higher incidence of desaturation and longer duration of emergence from anesthesia.^14^,^20^ In our study, remifentanil was used in almost all the patients and our findings were consistent with those reported in the literature.

Rocuronium, a non-depolarizing, short-acting neuromuscular blocker with a short half-life, causes minimal hemodynamic response and no histamine release. Rocuronium has been shown to significantly reduce the risk of laryngospasm and bronchospasm when used in combination with remifentanil.^21^ In our study, remifentanil was used in almost all the patients and the findings obtained were consistent with those reported in the literature. Succinylcholine is a depolarizing neuromuscular blocker which is commonly preferred for short surgical procedures, satiated patients, and patients requiring emergency intubation, mainly due to its rapid effects and short half-life. However, when administered in repeated doses, succinylcholine may result in rhabdomyolysis, cardiac arrest, and bradycardia.^22^ Won et al. reported that sugammadex led to faster reversal of rocuronium-induced neuromuscular blockade and shorter extubation time compared to atropine-neostigmine although no significant difference was found with regard to the incidence of adverse events following anesthetic induction.^23^ As consistent with the literature, in our study, reversal of rocuronium-induced neuromuscular blockade was achieved with atropine-neostigmine in 32.1% and sugammadex in 53.1% of the patients. On the other hand, succinylcholine was administered in 10 (12.3%) patients that underwent emergency bronchoscopy due to respiratory distress, although all the 10 patients were satiated. Side effects occurred in 5 (7.4%) patients that received rocuronium as opposed to 3 (30%) patients that received succinylcholine. Depending on these findings, it is safe to assert that rocuronium appears to be a better option than succinylcholine in patients planned for elective bronchoscopy under general anesthesia. In addition, no malignant hyperthermia, cardiac arrest, or aspiration occurred in any of our patients.

In a previous retrospective study, Hatipoglu et al. reported that almost 60% of their patients were transferred to ICU postoperatively and 10% of these patients required mechanical ventilation. In our study, however, although the need for mechanical ventilation was similar to that of the study by Hatipoglu et al. postoperative ICU admission was relatively lower.^4^ Another study reported that 6 (5.8%) patients required postoperative ICU admission due to sleepiness and sinuous arrhythmia (n=1), airway edema and the resulting respiratory distress (n=2), sleepiness and hypoxia (n=1), pneumonia and elongated aspiration (n=1), and respiratory distress and cyanosis (n=1).^10^ Williams et al. reported that delayed or missed treatment of desaturation, brachycardia, and bronchospasm may result in serious complications such as laryngeal edema, pneumothorax, and cardiac arrest. In patients with tracheobronchial FBA, steroids are recommended for the treatment of laryngeal edema.^24^ In our study, 18 (22.2%) were transferred to ICU and 63 (77.8%) patients were transferred to the ward postoperatively. Despite not being a cohort study, our study revealed that the incidence of laryngeal edema, laryngospasm, desaturation, and mucosal bleeding was significantly higher in the patients transferred to the ICU compared to those transferred to the ward (*p*<0.05). The rate of mortality associated with tracheobronchial FBA has been shown to be 0-1.8%.^2^ However, no mortality occurred in our patients.

### Limitations of the study

The study had a retrospective design. Further large-scale studies are needed to investigate the predisposing factors for postoperative ICU requirement in patients with tracheobronchial FBA.

## CONCLUSION

Bronchoscopy with general anesthesia remains the golden standard for the management of children with tracheobronchial FBA. We think that selection of short-acting anesthetic agents with minimal side effects for the induction and maintenance of anesthesia, and the administration of controlled ventilation can be recommended in children. Additionally, diagnosis of peri and post-operative complications, prediction of postoperative ICU requirement, and a close cooperation of anesthesiologists and surgeons are highly important.

### Authors’ Contribution:

**EK:** Study design, data collection and analysis, manuscript preparation and drafting.

**TY:** Study design, Review and final approval of manuscript.
